# Physical activity reduces hippocampal atrophy in elders at genetic risk for Alzheimer's disease

**DOI:** 10.3389/fnagi.2014.00061

**Published:** 2014-04-23

**Authors:** J. Carson Smith, Kristy A. Nielson, John L. Woodard, Michael Seidenberg, Sally Durgerian, Kathleen E. Hazlett, Christina M. Figueroa, Cassandra C. Kandah, Christina D. Kay, Monica A. Matthews, Stephen M. Rao

**Affiliations:** ^1^Department of Kinesiology, School of Public Health, University of MarylandCollege Park, MD, USA; ^2^Department of Psychology, Marquette UniversityMilwaukee, WI, USA; ^3^Department of Neurology, Medical College of WisconsinMilwaukee, WI, USA; ^4^Department of Psychology, Wayne State UniversityDetroit, MI, USA; ^5^Department of Psychology, Rosalind Franklin University of Medicine and ScienceNorth Chicago, IL, USA; ^6^Cleveland Clinic, Schey Center for Cognitive Neuroimaging, Neurological InstituteCleveland, OH, USA

**Keywords:** cognitive aging, Alzheimer's disease, volumetric MRI, association studies in genetics, physical activity, exercise

## Abstract

We examined the impact of physical activity (PA) on longitudinal change in hippocampal volume in cognitively intact older adults at varying genetic risk for the sporadic form of Alzheimer's disease (AD). Hippocampal volume was measured from structural magnetic resonance imaging (MRI) scans administered at baseline and at an 18-month follow-up in 97 healthy, cognitively intact older adults. Participants were classified as High or Low PA based on a self-report questionnaire of frequency and intensity of exercise. Risk status was defined by the presence or absence of the apolipoprotein E-epsilon 4 (APOE-ε4) allele. Four subgroups were studied: Low Risk/High PA (*n* = 24), Low Risk/Low PA (*n* = 34), High Risk/High PA (*n* = 22), and High Risk/Low PA (*n* = 17). Over the 18 month follow-up interval, hippocampal volume decreased by 3% in the High Risk/Low PA group, but remained stable in the three remaining groups. No main effects or interactions between genetic risk and PA were observed in control brain regions, including the caudate, amygdala, thalamus, pre-central gyrus, caudal middle frontal gyrus, cortical white matter (WM), and total gray matter (GM). These findings suggest that PA may help to preserve hippocampal volume in individuals at increased genetic risk for AD. The protective effects of PA on hippocampal atrophy were not observed in individuals at low risk for AD. These data suggest that individuals at genetic risk for AD should be targeted for increased levels of PA as a means of reducing atrophy in a brain region critical for the formation of episodic memories.

## Introduction

Possessing an apolipoprotein-E ε4 (APOE-ε4) allele increases the risk for developing the sporadic form of Alzheimer's disease (AD) (Bird, 2008). We have previously shown that APOE-ε4 status in healthy elders, in combination with measures of hippocampal atrophy obtained at study entry, can predict future cognitive decline after as short an interval as 18 months (Woodard et al., 2010). Such a finding is consistent with the commonly held view that the neuropathology of AD begins decades prior to the clinical diagnosis (Jack, 2012). Yet not all individuals with an APOE-ε4 allele will develop AD (Bird, 2008), suggesting that other genetic, and possibly lifestyle, factors may offer protection from neurodegeneration that ultimately leads to cognitive decline and a diagnosis of clinical AD.

Exercise training and physical activity (PA) are associated with the preservation cognitive function (Etnier et al., 2006; Angevaren et al., 2008) and hippocampal volume (Erickson et al., 2011) in healthy older adults. Whether APOE-ε4 status interacts with PA to slow the longitudinal course of neurodegeneration is unknown. Cross-sectional studies have reported differences between high and low PA on cognitive outcomes (Woodard et al., 2012; Smith et al., 2013), amyloid burden (Head et al., 2012), and brain function (Smith et al., 2011) in APOE-ε4 carriers (although see Lindsay et al., 2002). One such study (Schuit et al., 2001) reported reduced odds for experiencing cognitive decline in physically active older male APOE-ε4 carriers compared to sedentary carriers. Another study (Rovio et al., 2005) reported reduced odds of receiving an AD diagnosis in physically active male and female APOE-ε4 carriers compared to inactive carriers. Importantly, in both of these epidemiological studies, the positive effects of PA were not apparent in non-carriers. An additional epidemiological study (Lindsay et al., 2002) reported that engaging in regular PA reduced the odds of being diagnosed with AD after a 5 year follow-up period; in this study, however, the effects of PA were not modified by APOE-ε4 inheritance. Etnier and colleagues reported that greater cardiorespiratory fitness was associated with better neurocognitive test performance in healthy older women who were APOE-ε4 homozygotes, but the same relationship was not observed in APOE-ε4 heterozygotes or non-carriers. We (Woodard et al., 2012) have previously reported, in a sub-sample of the current study, that physically active APOE-ε4 carriers had significantly reduced odds of cognitive decline over 18 months compared to physically inactive carriers. However, it is not known if this pattern of neuroprotection among APOE-ε4 carriers also occurs in the hippocampus.

The aim of this study was to track the 18-month changes in hippocampal volume, a proxy for neurodegeneration, in four groups of cognitively intact older adults that varied on the basis of PA (low vs. high) and genetic risk (APOE-ε4 carriers vs. non-carriers). We hypothesized that high PA would result in preservation of hippocampal volume in APOE-ε4 carriers compared to low PA carriers. In contrast, the protection offered by PA would be less apparent in APOE-ε4 non-carriers.

## Materials and methods

### Participants

Healthy adults between the ages of 65 and 89 were recruited from newspaper advertisements. A telephone screen was administered initially to 459 individuals to determine eligibility based on inclusion/exclusion criteria. Potential participants were excluded if they reported a history of cognitive deterioration and/or dementia, neurological disease (cerebral ischemia, vascular headache, carotid artery disease, cerebral palsy, epilepsy, brain tumor, chronic meningitis, multiple sclerosis, pernicious anemia, normal-pressure hydrocephalus, HIV infection, Parkinson's disease, and Huntington's disease), medical illnesses (untreated hypertension, glaucoma, and chronic obstructive pulmonary disease), major psychiatric disturbance or substance abuse generally consistent with DSM-IV Axis I criteria or a Geriatric Depression Scale (GDS) score greater than 15. Participants were allowed to take cardiovascular drugs.

To enrich the sample with a higher percentage of APOE-ε4 carriers, half the sample was recruited based on a family history of dementia, since the APOE-ε4 allele is more common in individuals with a family history of dementia than among those without such a history (Sager et al., 2005). Family history was defined as a report of a clear clinical diagnosis of AD or a reported history of gradual decline in memory and other cognitive functions, confusion, or judgment problems without a formal diagnosis of AD prior to death in a first-degree relative.

Of those who met criteria, 112 agreed to undergo APOE genotyping from blood samples, complete a PA questionnaire, and be administered a brief neurobehavioral assessment and a structural magnetic resonance imaging (MRI) scan. All participants were invited to undergo a repeat MRI scan 18 months later. Follow-up MRI scans were obtained from 99 of 112 (88.4%) participants using the identical scanner and pulse sequence (see below). Of the 13 participants who did not undergo follow-up scanning, nine withdrew from the study, two had excessive head motion at the baseline scan, and two were lost to follow-up. Of the 99 participants who completed both scan sessions, two were excluded due to excessive head motion during follow-up scanning. From the final pool of 97 participants, four subgroups were formed based on the absence/presence of one or both APOE-ε4 alleles (Low Risk vs. High Risk) and self-reported amounts of leisure-time PA (Low PA vs. High PA). The mean (±*SD*) inter-scan interval for all participants was 554 (±41) days (range 489–763); the mean inter-scan interval did not differ between groups (*p* = 0.92).

This study was approved by the institutional review board at the Medical College of Wisconsin and conducted in accordance with the Helsinki Declaration. Written informed consent was obtained and all participants received modest financial compensation.

### Physical activity

Frequency and intensity of leisure time PA were measured using the Stanford Brief Activity Survey (SBAS) (Taylor-Piliae et al., 2006). SBAS scores have demonstrated validity for assessing habitual PA (Taylor-Piliae et al., 2006, 2007). Participants who endorsed one of the two items indicating two or fewer days of low intensity PA (ranging from no PA to slow walking or light chores) were classified as physically inactive (Low PA). Participants endorsing one of the remaining three items describing moderate to vigorous intensity PA three or more days per week (ranging from brisk walking, jogging or swimming for 15 min or more, or moderately difficult chores for 45 min, to regular jogging, running, bicycling or swimming for 30 min or more, or playing sports such as handball or tennis for an hour or more) were classified as physically active (High PA).

### Genetic testing

APOE genotype was determined using a polymerase chain reaction method (Saunders et al., 1996). Deoxyribonucleic acid was isolated with Gentra Systems Autopure LS for Large Sample Nucleic Acid Purification. Participants with one or both APOE-ε4 alleles were classified as High Risk for developing AD and the remainder classified as Low Risk. APOE genotype results for the four groups were as follows: Low Risk/High PA (*n* = 24: three ε2/ε3 and 21 ε3/ε3), Low Risk/Low PA (*n* = 34: six ε2/ε3 and 28 ε3/ε3), High Risk/High PA (*n* = 22: 20 ε3/ε4 and two ε4/ε4), and High Risk/Low PA (*n* = 17: two ε2/ε4 and 15 ε3/ε4).

### Neurobehavioral testing

Participants were administered the Mini-Mental State Examination (Folstein et al., 1975), Mattis Dementia Rating Scale 2 (DRS-2) (Jurica et al., 2001), GDS (Yesavage, 1988), and Lawton Activities of Daily Living (ADL) (Lawton and Brody, 1969) at study entry.

### Structural MRI acquisition

MR imaging was conducted at baseline and 18-month follow-up on a General Electric (Waukesha, WI) Signa Excite 3T short bore scanner equipped with a quad split quadrature transmit/receive head coil. High-resolution, 3-dimensional spoiled gradient-recalled at steady state (SPGR) anatomic images were acquired [*TE* 3.9 ms; repetition time (*TR*) 9.5 ms; inversion recovery preparation time 450 ms; flip angle 12°; number of excitations 2; slice thickness 1.0 mm; FOV 24 cm; resolution 256 × 224]. Foam padding was used to reduce head movement.

### Volumetric analyses

The hippocampus was selected *a priori* as the key structure for examining the longitudinal volumetric effects of PA. In addition, we measured changes in volumes of the thalamus, caudate, amygdala, caudal middle frontal gyrus, pre-central gyrus, total gray matter (GM), and cortical white matter (WM) to determine if PA effects were specific to the hippocampus. Volumetric measurements were obtained from parcellation of anatomical T1 images at baseline and at 18-months using the longitudinal method of FreeSurfer software (v. 5.1) (Fischl et al., 2004). The first stage consisted of identifying the GM/WM boundary (Dale et al., 1999) using both intensity and continuity information from the entire 3D volume. Maps were then created using spatial intensity gradients across tissue classes. Using an automated labeling system, GM and WM were subdivided into distinct volumes of interest per hemisphere (Desikan et al., 2006). Homologous left and right hemisphere volumes were summed, as these volumes were highly correlated and we did not have a hypothesis regarding hemispheric differences. In addition, total intracranial volume (ICV) was calculated to account for inter-individual differences in head size. Each volume of interest was expressed as a percentage of total ICV (%ICV). Change in volume was achieved by subtracting baseline and follow-up %ICV and then expressed as a percent change from baseline. This change score was subjected to a 2 (High Risk vs. Low Risk) × 2 (High PA vs. Low PA) analysis of variance (ANOVA; SPSS 21, Chicago, IL). Significant effects were followed by *post-hoc* group comparisons.

## Results

### Baseline analyses

The four participant groups did not differ in age, education, or sex at baseline (see Table 1). There were no differences in the number of participants in each group based on PA and Risk classification (*p* > 0.2). At baseline, the High Risk groups performed slightly worse on the MMSE than the Low Risk groups, but all study participants performed within the normal range indicating intact cognitive abilities. No significant group differences were observed on a measure of depression (GDS) and all participants had intact activities of daily living. At baseline, no significant main or interaction effects were observed for %ICV for the hippocampus, thalamus, caudate, caudal middle frontal gyrus, and cortical WM. A significant PA effect was observed for the amygdala, pre-central gyrus, and total GM (Low PA > High PA).

**Table 1:** **Mean (*SD*) baseline demographic, behavioral testing, and brain volume measures for the four participant groups**.

**Variables**	**Low risk**		**High risk**		**ANOVA**					
	**High PA**	**Low PA**	**High PA**	**Low PA**	**Risk**		**PA**		**Interaction**	
	**(*n* = 24)**	**(*n* = 34)**	**(*n* = 22)**	**(*n* = 17)**	***p***	**η**^2^_*p*_****	***p***	**η **^2^_*p*_****	***p***	**η**^2^_*p*_****
**DEMOGRAPHICS**										
Age (years)	74.4 (5.2)	72.2 (4.6)	71.5 (4.2)	73.7 (5.5)	0.489	0.005	0.952	<0.001	**0.033**	**0.048**
Education (years)	15.0 (2.8)	14.0 (2.0)	15.5 (3.0)	15.6 (3.0)	0.088	0.024	0.461	0.006	0.329	0.010
Sex	9 M, 15 F	7 M, 27 F	5 M, 17 F	7 M, 10 F	–	–	–	–	0.300^*^	0.038
**BEHAVIORAL TESTING**										
MMSE	29.4 (0.8)	29.4 (0.7)	29.2 (1.3)	28.6 (1.2)	**0.013**	**0.064**	0.207	0.017	0.115	0.026
DRS total score	140.0 (3.8)	141.0 (2.3)	139.7 (3.3)	139.2 (4.7)	0.135	0.024	0.768	0.001	0.318	0.011
GDS	1.7 (2.0)	2.8 (2.7)	2.5 (3.0)	2.2 (2.6)	0.854	<0.001	0.449	0.006	0.205	0.017
**BRAIN VOLUME**										
Hippocampus	0.456 (0.061)	0.477 (0.080)	0.447 (0.065)	0.460 (0.073)	0.386	0.008	0.263	0.013	0.781	0.001
Thalamus	0.789 (0.062)	0.829 (0.091)	0.800 (0.090)	0.826 (0.093)	0.820	0.001	0.071	0.035	0.678	0.002
Caudate	0.504 (0.063)	0.542 (0.067)	0.499 (0.063)	0.508 (0.063)	0.152	0.022	0.091	0.030	0.285	0.012
Amygdala	0.178 (0.024)	0.191 (0.028)	0.177 (0.027)	0.187 (0.026)	0.597	0.003	**0.034**	**0.047**	0.741	0.001
Caudal middle frontal g.	0.834 (0.108)	0.815 (0.125)	0.823 (0.126)	0.828 (0.129)	0.970	<0.001	0.765	0.001	0.664	0.002
Pre-central g.	1.76 (0.15)	1.82 (0.18)	1.73 (0.17)	1.85 (0.17)	0.936	<0.001	**0.015**	**0.062**	0.387	0.008
Total GM	38.6 (2.8)	40.4 (4.1)	38.6 (2.5)	40.6 (3.0)	0.875	<0.001	**0.008**	**0.074**	0.871	<0.001
Cortical WM	30.5 (2.8)	31.2 (3.4)	30.2 (3.5)	30.6 (2.6)	0.508	0.005	0.398	0.008	0.823	0.001

Bold font indicates significant effect; η^2^_*p*_, partial eta-squared, a measure of effect size.

* *Based on Chi-Square statistic; MMSE, Mini-Mental State Exam; DRS, Dementia Rating Scale; GDS, Geriatric Depression Scale; g., gyrus; GM, gray matter; WM, white matter*.

### Longitudinal change in brain volumes

**Figure 1:**
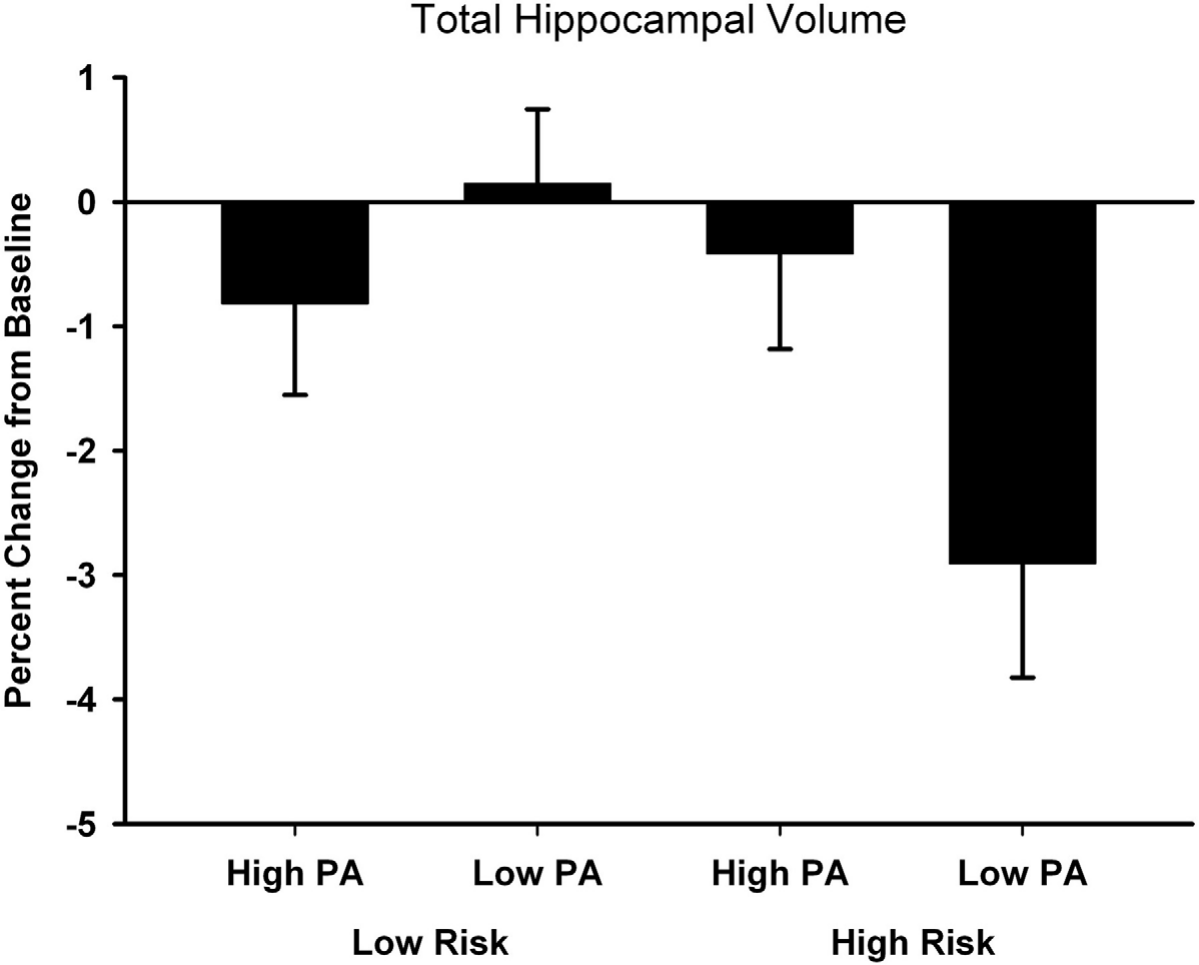
**Percent change from baseline in total hippocampal volume for the four participant groups**. Error bars represent s.e.m.

The percent change from baseline to 18-month follow-up scans are shown in Table 2 (see Table 3 for raw volumetric data). A significant interaction was observed between Risk and PA for the hippocampus. Hippocampal volume decreased 3% in the High Risk/Low PA group (see Figure 1), whereas the volumetric changes in the remaining three groups were negligible. No significant main or interaction effects were observed for the thalamus, caudate, amygdala, caudal middle frontal gyrus, pre-central gyrus, total GM, and cortical WM volumes.

**Table 2:** **Change in brain volumes after 18 months expressed as mean (*SD*) percent change from baseline**.

**Brain volume**	**Low risk**		**High risk**		**ANOVA**					
	**High PA**	**Low PA**	**High PA**	**Low PA**	**Risk**		**PA**		**Interaction**	
	**(*n* = 24)**	**(*n* = 34)**	**(*n* = 22)**	**(*n* = 17)**	***p***	**η **^2^_*p*_****	***p***	**η**^2^_*p*_****	***p***	**η **^2^_*p*_****
Hippocampus	−0.82 (3.60)	0.15 (3.49)	−0.41 (3.61)	−2.91 (3.79)	0.082	0.032	0.314	0.011	**0.024**	**0.054**
Thalamus	−2.06 (2.72)	−0.85 (3.05)	−1.77 (2.23)	−1.61 (2.32)	0.677	0.002	0.228	0.016	0.351	0.009
Caudate	−0.48 (3.58)	−1.24 (3.69)	−1.23 (2.73)	−2.44 (3.42)	0.177	0.020	0.171	0.020	0.759	0.001
Amygdala	0.59 (7.68)	1.40 (8.40)	−0.10 (6.86)	−0.17 (4.77)	0.465	0.006	0.812	0.001	0.777	0.001
Caudal middle frontal g.	0.92 (3.42)	1.04 (3.95)	0.11 (4.74)	0.89 (5.08)	0.593	0.003	0.614	0.003	0.715	0.001
Pre-central g.	−0.09 (3.45)	−0.51 (4.31)	−0.97 (4.68)	−1.21 (4.48)	0.375	0.008	0.711	0.001	0.924	<0.001
Total GM	−0.39 (2.27)	−0.49 (3.13)	−0.26 (3.27)	−1.37 (3.18)	0.551	0.004	0.340	0.010	0.421	0.007
Cortical WM	−1.43 (2.66)	−0.37 (2.07)	−0.65 (1.42)	−0.56 (1.37)	0.486	0.005	0.178	0.019	0.252	0.014

*Bold font indicates significant effect; η^2^_*p*_, partial eta-squared; PA, physical activity; GM, gray matter; WM, white matter; g., gyrus*.

**Table 3:** **Absolute mean (*SD*) brain volume at baseline and 18-months expressed as a percent of total intracranial volume**.

**Brain volume**	**Low risk**		**High risk**	
	**High PA**	**Low PA**	**High PA**	**Low PA**
	**(*n* = 24)**	**(*n* = 34)**	**(*n* = 22)**	**(*n* = 17)**
**HIPPOCAMPUS**				
Baseline	0.456 (0.061)	0.480 (0.081)	0.447 (0.065)	0.460 (0.073)
18 months	0.451 (0.059)	0.478 (0.083)	0.445 (0.069)	0.447 (0.078)
**THALAMUS**				
Baseline	0.789 (0.062)	0.829 (0.091)	0.800 (0.090)	0.826 (0.093)
18 months	0.773 (0.070)	0.822 (0.093)	0.787 (0.099)	0.811 (0.085)
**CAUDATE**				
Baseline	0.504 (0.063)	0.542 (0.067)	0.499 (0.063)	0.508 (0.063)
18 months	0.502 (0.066)	0.535 (0.070)	0.493 (0.061)	0.496 (0.065)
**AMYGDALA**				
Baseline	0.178 (0.024)	0.191 (0.028)	0.177 (0.027)	0.187 (0.026)
18 months	0.178 (0.025)	0.194 (0.033)	0.176 (0.030)	0.187 (0.029)
**CAUDAL MIDDLE FRONTAL g**.				
Baseline	0.834 (0.108)	0.815 (0.125)	0.823 (0.126)	0.828 (0.129)
18 months	0.842 (0.115)	0.824 (0.137)	0.824 (0.130)	0.836 (0.143)
**PRECENTRAL g**.				
Baseline	1.76 (0.15)	1.82 (0.18)	1.73 (0.17)	1.85 (0.17)
18 months	1.76 (0.13)	1.81 (0.21)	1.72 (0.18)	1.83 (0.21)
**TOTAL GM**				
Baseline	38.6 (2.8)	40.4 (4.1)	38.6 (2.5)	40.6 (3.0)
18 months	38.5 (2.8)	40.2 (4.3)	38.5 (3.0)	40.1 (3.2)
**CORTICAL WM**				
Baseline	30.5 (2.8)	31.2 (3.4)	30.2 (3.5)	30.6 (2.6)
18 months	30.1 (3.2)	31.1 (3.5)	30.0 (3.5)	30.5 (2.6)

*PA, physical activity; GM, gray matter; WM, white matter; g., gyrus*.

## Discussion

Atrophy of the hippocampus is a hallmark of AD progression and, to a lesser extent, occurs as part of healthy aging. We have demonstrated that the rate of hippocampal atrophy can be influenced by the extent of leisure-time PA. The protective effect of PA on hippocampal volume, however, was specific to persons at genetic risk for AD and was not observed in individuals at low genetic risk. Remarkably, the protective effect of PA in high risk individuals was seen over a relatively brief 18 month follow-up interval. Our findings were found to be specific to the hippocampus, since similar PA effects were not observed in the thalamus, caudate, amygdala, caudal middle frontal gyrus, pre-central gyrus, total GM, or cortical WM volumes.

This study is the first to demonstrate the protective effects of PA on hippocampal atrophy in persons at genetic risk for AD. Our results are compatible with epidemiological studies that have examined the joint effects of PA and APOE-ε4 inheritance on the extent of cognitive decline and the diagnosis of AD (Smith et al., 2013). Our findings are also consistent with evidence from other brain imaging modalities. Head and colleagues, using [^11^C] Pittsburgh Compound B (PiB) as a measure of amyloid mean cortical binding potential, reported less brain amyloid in a physically active compared to a physically inactive group of APOE-ε4 carriers, an effect not observed in non-carriers. Deeny et al. (2012) demonstrated that among ε4 carriers, activation in the left inferior temporal cortex during performance of the Sternberg working memory task was greater in those with higher levels of fitness. Finally, in a task-activated fMRI study conducted on a sub-sample of this study, we (Smith et al., 2011) demonstrated that semantic memory activation was greatest in high PA APOE-ε4 carriers relative to low PA carriers and non-carriers. We have also demonstrated that increased semantic memory activation, along with APOE-ε4 non-carrier status, is protective against future cognitive decline (Woodard et al., 2010). In a sub-sample of the current study, physically active APOE-ε4 carriers had significantly reduced odds of cognitive decline over 18 months compared to physically inactive carriers (Woodard et al., 2012); as in the current study of hippocampal volume, the protective effect of PA was not observed in APOE-ε4 non-carriers. Other factors, such as the presence of depressive symptoms, may also lead to reductions in cognitive function and hippocampal volume in older adults (Sexton et al., 2013). However, in our sample depression scores were below the clinical range, did not differ between the groups at baseline, and did not significantly change at the 18-month follow-up. Thus, these effects do not appear to be related to differences in symptoms of depression between the groups.

### Potential mechanisms for physical activity and APOE-ε4 interactions

The precise neurophysiological mechanisms by which PA might protect human APOE-ε4 carriers from cognitive decline and AD-related neuropathology are less well understood (Smith et al., 2013). Animal studies would suggest that PA counteracts the physiologic impact of the APOE-ε4 allele, possibly through benefits to cholinergic function or brain lipid metabolism, and/or reduced neuroinflammation (for a review, see Intlekofer and Cotman, 2013). Physical activity is known to promote the release of neurotrophins [brain derived neurotrophic factor (BDNF), insulin-like growth factor-1] that support neurogenesis in the dentate gyrus (Trejo et al., 2001; Van Praag et al., 2005). In addition, cholinergic enhancement due to PA may increase cerebral blood flow, enhance neural activation, and possibly relieve amyloid burden (Adlard et al., 2005; Head et al., 2012). In humans, recent evidence suggests that PA may have similar neurogenic effects in the hippocampi of healthy younger adults (Pereira et al., 2007) and healthy older adults (Erickson et al., 2011). However, it is not known if the neurotrophic effects of PA are stronger among APOE-ε4 carriers.

Because inheriting an APOE-ε4 allele results in the disruption of lipid homeostasis, there are adverse effects on amyloid precursor protein (APP) function and the clearance of brain amyloid, as well as on neuroinflammation and acetylcholine function (Poirier, 2000; Lane and Farlow, 2005). Apolipoproteins are lipid carrying molecules involved in regulating lipid metabolism in response to neuronal injury. The protein APOE is particularly important in brain synaptic plasticity and growth through its role handling phospholipids and cholesterol associated with neuronal repair processes (Poirier, 2000). Lipoproteins resulting from the APOE-ε4 allele are removed more easily in carriers, and this greatly reduces the amount of APOE available in the brain compared to non-carriers (Leduc et al., 2011). A concomitant decrease in lipoprotein lipase activity also leads to reduced levels of brain free fatty acids, fundamental components of neuronal repair and neurotrophic processes (Lane and Farlow, 2005), which alters APP function to further the production and accumulation of brain β-amyloid (Poirier, 2000). Finally, altered lipid membrane homeostasis reduces glycolytic metabolic processes and the availability of acetyl-CoA-derived adenosine triphosphate and acetylcholine (Lane and Farlow, 2005; Leduc et al., 2011), leading to compromised cholinergic function. Exercise has been shown to improve cholinergic function and to oppose the actions of acetylcholinesterase in the hippocampus and cerebral cortex of rats (Ben et al., 2009).

A small number of animal studies have reported interactions between APOE genotype and exercise on brain neurophysiology. APOE-ε4 mice showed similar increases in hippocampal BDNF compared to APOE-ε3 mice after 6 weeks of voluntary wheel running using a transgenic mouse model. Moreover, tyrosine kinase B receptors, which have a high affinity for BDNF, increased after wheel running in APOE-ε4 mice to levels comparable to the baseline levels of APOE-ε3 mice (Nichol et al., 2009). Exercise in APOE-lacking mice may attenuate the development of atherosclerosis in the periphery, and these effects may be due to exercise-induced enhancement of anti-inflammatory cytokines (Fukao et al., 2010). The effects of PA on brain lipid metabolism in human ε4 carriers are not yet known (Rankinen et al., 2010), nor if responses to exercise training differ based on APOE genotype (Leon et al., 2004). The beneficial effects of PA are not exclusive, however, to APOE. For example, exercise has been shown to improve cognition, reduce oxidative stress, and induce synaptic plasticity in the 3xTg-AD triple transgenic mouse model of AD (Garcia-Mesa et al., 2011), underscoring its pleiotropic effects. To date, the literature suggests that physical *inactivity* may exacerbate the effects of the APOE-ε4 genotype on AD-related neuropathology and its clinical manifestation of memory impairment (Smith et al., 2013), consistent with the current findings.

### Limitations

It is important to note that we did not manipulate levels of PA nor did we randomly assign participants to groups in a controlled trial, thus limiting our ability to determine cause-effect relationships. We also did not control for other health-related behaviors (e.g., diet) or measure BDNF genotype. The sample was mostly Caucasian (only one Hispanic and one African-American participated), so these results may not generalize to other ethnic groups. This study is also limited by a subjective measure of leisure-time PA rather than an objective measurement of cardiorespiratory fitness, such as the maximal rate of oxygen consumption (VO_2max_). Responses to the SBAS, however, were dose-dependently related to cardiovascular risk biomarkers and estimated caloric expenditure in an epidemiologic study (Taylor-Piliae et al., 2006). The High Risk/Low PA group included two participants with the ε2ε4 genotype. Although the APOE-ε2 allele has been associated with protection from morbidity, results from the Rotterdam Study (Slooter et al., 1998) indicate that this effect is clearest for the ε2ε3 genotype, with a greater risk of cognitive impairment for the ε2ε4 genotype.

### Conclusions

Our study has provided additional evidence that PA can afford protection against neurodegeneration in cognitively intact persons at genetic risk for AD. Our results suggest that knowledge of the APOE genotype, while hardly precise in the prediction of AD, can play an important role in making recommendations to older adults regarding exercise as a means of maintaining brain integrity and preventing future cognitive decline and brain atrophy. Future studies are needed to better understand the neurophysiological mechanisms by which PA appears to alter the phenotypic expression of the APOE-ε4 allele.

## Author contributions

J. Carson Smith, Kristy A. Nielson, John L. Woodard, Michael Seidenberg, and Stephen M. Rao contributed to the design and conceptualization of the study, the analysis and interpretation of the data, and drafting and revising the manuscript for intellectual content. Sally Durgerian, Kathleen E. Hazlett, Christina M. Figueroa, Cassandra C. Kandah, Christina D. Kay, and Monica A. Matthews, contributed to the analysis and interpretation of the data and drafting and revising the manuscript for intellectual content.

### Conflict of interest statement

The authors declare that the research was conducted in the absence of any commercial or financial relationships that could be construed as a potential conflict of interest.
